# Optimization of Microfluidics for Point-of-Care Blood Sensing

**DOI:** 10.3390/bios14060266

**Published:** 2024-05-23

**Authors:** Amirmahdi Tavakolidakhrabadi, Matt Stark, Ulrike Bacher, Myriam Legros, Cedric Bessire

**Affiliations:** 1 Department of Engineering and Computer Science, Bern University of Applied Sciences, Quellgasse 21, 2501 Biel, Switzerland; amirmahdi.tavakolidakhrabadi@bfh.ch (A.T.); matt.stark@bfh.ch (M.S.); 2 Department of Hematology and Central Hematology Laboratory, Inselspital, Bern University Hospital, 3010 Bern, Switzerland; veraulrike.bacher@insel.ch (U.B.); myriam.legros@insel.ch (M.L.)

**Keywords:** biomedical sensors, lab-on-a-chip devices, capillary-driven systems, microscale fluid transport, computational modeling, microfluidics, point-of-care blood sensing

## Abstract

Blood tests are widely used in modern medicine to diagnose certain illnesses and evaluate the overall health of a patient. To enable testing in resource-limited areas, there has been increasing interest in point-of-care (PoC) testing devices. To process blood samples, liquid mixing with active pumps is usually required, making PoC blood testing expensive and bulky. We explored the possibility of processing approximately 2 μL of whole blood for image flow cytometry using capillary structures that allowed test times of a few minutes without active pumps. Capillary pump structures with five different pillar shapes were simulated using Ansys Fluent to determine which resulted in the fastest whole blood uptake. The simulation results showed a strong influence of the capillary pump pillar shape on the chip filling time. Long and thin structures with a high aspect ratio exhibited faster filling times. Microfluidic chips using the simulated pump design with the most efficient blood uptake were fabricated with polydimethylsiloxane (PDMS) and polyethylene oxide (PEO). The chip filling times were tested with 2 μL of both water and whole blood, resulting in uptake times of 24 s for water and 111 s for blood. The simulated blood plasma results deviated from the experimental filling times by about 35% without accounting for any cell-induced effects. By comparing the flow speed induced by different pump pillar geometries, this study offers insights for the design and optimization of passive microfluidic devices for inhomogenous liquids such as whole blood in sensing applications.

## 1. Introduction

Blood testing plays a vital role in medical diagnosis, treatment, and health monitoring [1]. A shift towards PoC testing has been seen over the last few years [2] due to its quicker, more affordable, and more convenient testing [3]. PoC is advantageous in making quick medical decisions and helps to diagnose diseases at an early stage or monitor changes during a healing phase [4]. If the appropriate decisions are subsequently taken, better and faster recovery outcomes for patients result, leading to an overall lower medical burden and cost for society [5]. The difficulty with PoC blood testing is the processing of blood without any external energy resources for pumping and thus a constant blood flow in order to make the testing reliable, rapid, and robust [6]. These are just a few of the required properties for PoC testing, according to the World Health Organization (WHO) [7], that need to be fulfilled for low-resource settings or areas with limited laboratory access [8]. Capillary action refers to the remarkable ability of a liquid to flow through narrow spaces or channels without external forces such as gravity [9]. Thus, for analyte testing in liquids or blood, capillary drawing by nitrocellulose structures is the state of the art [10]. However, if cells need to be analyzed at the PoC, capillary action must be present in larger microfluidic channels [11]. The challenge for blood cell PoC applications is not only to ensure capillary action but to generate a strong, non-varying pumping effect in order to process the cells efficiently within a few minutes to generate a quick PoC result [12,13].

In PoC diagnostics, the state of the art in passive capillary pumping uses nitrocellulose substrates [14,15,16]. While effective for plasma processing, they filter out cellular components, limiting them to plasma-based analysis. Active pump systems offer an alternative by enabling whole blood processing [17,18,19]; however, they introduce complexity and costs that may not be justifiable in certain settings, particularly in PoC applications. Addressing these limitations, our research introduces a passive device designed to match the processing capabilities of nitrocellulose-based systems while enabling cell analysis, offering a simpler, cost-effective solution for comprehensive blood diagnostics. Research has been conducted on improving the capillary action in biocompatible polymer microfluidic systems [20,21,22,23,24,25,26,27,28,29,30,31]. Certain systems have been maximized in terms of the pumping efficiency without using nitrocellulose capillary pump structures [23,32]. However, these systems have not been tested with whole blood. Therefore, we present, in this study, further design possibilities for capillary pump structures. These were investigated in Ansys simulations to find the most efficient capillary pumping design, which was subsequently experimentally verified with blood plasma as well as whole blood.

**Figure 1:**
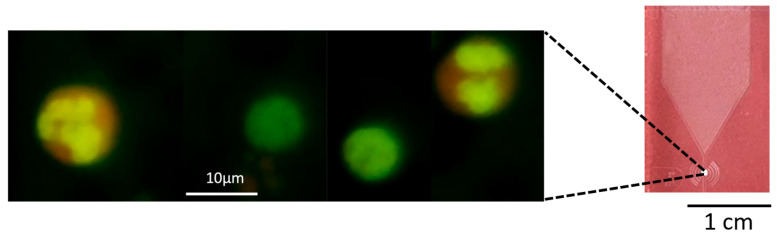
White blood cells stained with Acridine Orange in a microfluidic channel. The channel is epi-illuminated with a 455 nm LED, causing the two visible lymphocytes to fluoresce green and the two granulocytes to fluorescence green and red. Capillary forces move the cells through the imaging field of view, as demonstrated in the actual image of the microfluidic chip.

In the context of microfluidic chips employed at the PoC, capillary action plays a pivotal role in order to generate reliable medical fluid testing. For the purpose of sensing cells, we stained white blood cells and imaged them accordingly [33], as can be seen in Figure 1. This approach presents several benefits, including the ability to count cells and identify irregularities in cell shape and structure. The counting of white blood cells is pivotal in diagnosing a range of infections and medical conditions [34], while detecting abnormalities in cell morphology can offer valuable information about a patient’s overall health and potential diseases [35,36]. Consequently, the incorporation of microfluidic systems driven by capillary action in PoC testing holds significant potential in improving the speed, effectiveness, and diagnostic capacity of blood testing across various clinical scenarios [37] and thus improving healthcare by lowering costs [38]. 

By utilizing capillary forces, the chip facilitates the controlled manipulation and movement of the blood within its intricate microchannels [39]. Consequently, whole blood samples can be accurately positioned in the focal plane, optimizing the acquisition of detailed microscopic images, as can be seen in Figure 1. This is only one application out of several that require efficient pumping for quick PoC testing by leveraging cost-effective passive microfluidics technology, eliminating the need for external carrier fluids or cumbersome pumps [40]. An efficient capillary pump offers a versatile platform for a wide range of diagnostic possibilities, including optical, electrical, biological, and antibody-based assays, enabling the comprehensive measurement of various blood and health parameters including cells at the PoC [41,42].

In this context, this research delves into the examination of capillary pump configurations that have undergone rigorous simulation and experimental evaluation, with a unique emphasis on allowing the transit of various blood cell types, encompassing white blood cells. This innovative approach not only serves to enhance the analyte detection capabilities but also highlights the potential for the real-time monitoring and control of diverse cell types directly at the PoC, significantly expanding the scope and possibilities of PoC testing in the realm of diagnostics [22].

## 2. Materials and Methods

The microfluidic design considerations, the simulation details, and the fabrication of the PDMS chips used in the experiments are discussed in the following sections.

### 2.1. Microchannel Geometry

**Figure 2:**
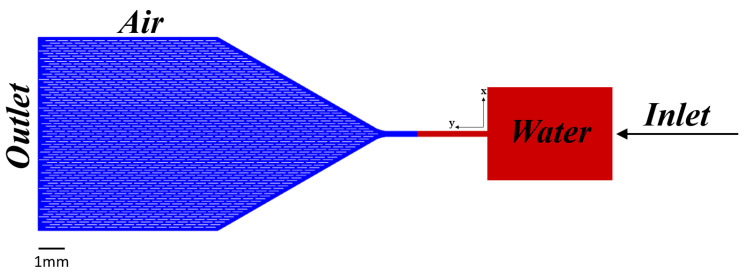
Microfluidic chip schematic. A liquid sample is placed on the rectangular inlet on the right. Capillary forces draw the liquid through a 250 μm × 30 μm channel before entering the capillary pump on the left. Red indicates the 25% liquid volume in the chip used as an initial condition for the simulations.

**Figure 3:**
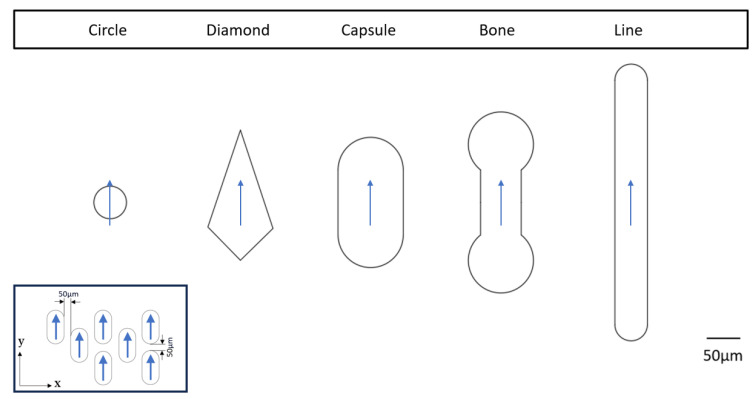
Pillar shapes used in capillary pump simulations; from left to right: circle, diamond, capsule, bone, and line. The arrows on each capillary pump indicate the alignment direction of the pumps on the microfluidic chip. Additionally, the diagram within the box illustrates the arrangement of the pillars in relation to each other along the *x* and *y* axes. Within the capillary pump, neighboring pillars are separated by 50 μm in the *x* and *y* directions.

The microchannel geometry is illustrated in Figure 2. A rectangular inlet pad is connected to a capillary pump via a 250 μm wide × 30 μm deep channel where cell images are captured. The channel width is limited by the field of view of the microscope. The capillary pump is filled with one of the specific pillar shapes shown in Figure 3, arranged in an offset grid with 50 μm wall-to-wall separation in both directions. The conical inlet shape in the design serves several purposes. Firstly, it enhances the pump filling efficiency. Secondly, it ensures a gradual increase in the overall speed of the system. This gradual speed transition is essential because a sudden velocity surge would pose challenges in terms of cell imaging or sensing in the main channel.

### 2.2. Governing Equations

To analyze the capillary flow within the microchannel, transient and three-dimensional numerical simulations were conducted using the Volume of Fluid (VOF) method [43]. The system comprises two fluids: a liquid phase, which is, in our case, water or blood plasma, and a gas phase, namely air, both treated as incompressible and immiscible [44]. This simplification, assuming the gas phase, like air, to be incompressible, can be made for microfluidic simulations, since the compressibility effects of the gas phase compared to the liquid phase are negligible [45]. Despite gases being approximately 20,000 times more compressible than water in bulk [46], at microscale levels, the effects of compressibility in the gas phase are negligible [47]. This is primarily due to the extremely small volumes and high surface-to-volume ratios in microfluidic systems, where the gas’s compressibility becomes less significant and can be effectively ignored [48] for practical simulation purposes. This simplification allows for accurate results and streamlined simulations, as the higher density and dominance of the liquid phase in microfluidic systems justifies treating both phases as incompressible.

The VOF method incorporates surface tension effects, which are paramount for capillary forces, and we calculate their effects by solving the momentum and continuity equations in the simulations [49].

At low velocities, blood plasma can be considered as a Newtonian fluid within the context of microchannels [50]. Newtonian fluids exhibit a linear relationship between the shear stress and shear rate, meaning that the viscosity remains constant regardless of the applied shear [51]. This assumption allows for the simplified mathematical modeling and analysis of the blood plasma flow dynamics within the microchannel. This simplification holds some validity in our microfluidic channel scenario, mainly because we consider low velocities in the range of 0.1–20 mm/s. Thus, the simulation reflects blood plasma but not whole blood’s capillary filling behavior. Furthermore, the flow is assumed to be laminar, incompressible, Newtonian, and isothermal, as the experiments were all conducted at room temperature. The velocity field is governed by the Navier–Stokes and continuity equations.

The surface tension model used in this study is based on the continuum surface force (CSF) model, originally proposed by Brackbill et al. [52], calculated as

(1) $$F_{s}=\sigma \kappa \nabla F$$

where σ is the surface tension coefficient, κ the surface curvature, and ∇F the normal vector. In the CSF model, the surface tension is assumed to remain constant across the surface [53]. Only the forces perpendicular to the interface are taken into account.

#### Initial Boundary Conditions

In the initial moment (t=0 s), the fluid occupies 25% of the total volume, as depicted in Figure 2. This initial condition was set to mirror the real-world dynamics, where it typically takes around 5s for the water to naturally fill the resource area and flow into the main chip with its embedded pillars. In our simulation, the time taken for the fluid to reach the chip with the pillars is considerably longer than 5 s. This extended duration arises from our deliberate choice not to introduce high-velocity flow conditions, which would deviate from our underlying simulation assumptions. These assumptions are rooted in the idea that, under low-velocity conditions, the flow remains smooth and orderly. Conversely, at higher velocities, the flow tends to become turbulent and chaotic. Therefore, pre-filling the resource area and channel was necessary to enhance the alignment between our simulation and real-world scenarios, ensuring the fidelity of our modeling and results. At the core of our research, the focal point was within the main chip adorned with its intricate array of pillars. Understanding the precise role and functionality of these pillars within the main chip constituted the most critical aspect of our investigation. The boundary condition at the inlet was set as a velocity inlet of 0.1 mm/s. The outlet boundary condition was defined as a pressure outlet—specifically, a gauge pressure of zero.

The walls of the channel are subjected to a no-slip boundary condition, meaning that the fluid velocity is zero at the walls [54]. The surface affinity of the walls is defined by the contact angles, which characterize the interaction between the fluid and the surface. In some simulation scenarios, a contact angle value of zero is assigned to all wall surfaces, signifying that the fluid completely wets the walls and does not form any droplets [55]. However, in our specific case, we observed in our real-world measurements that polyethylene oxide (PEO)–polydimethylsiloxane (PDMS) exhibits contact angles ranging within a few degrees, typically between 1 and 5 degrees. This observation signifies that the fluid does not fully wet the walls; instead, it tends to bead up slightly on the surface, forming small droplets or exhibiting partial wetting behavior. In essence, this means that the fluid–surface interaction in our system is not characterized by complete wetting, which has implications for the behavior of the fluid within the microfluidic structure and influences its overall performance.

The physical properties of the air, water, and blood plasma used for the simulations are listed in Table 1, with all fluids assumed to be homogeneous.

### 2.3. Microchannel Fabrication

Microchannel master casting structures were created via photo-lithography on a silicon wafer [56], with the final chip fabrication performed using PDMS and soft lithography [57]. A uniform layer of a 30 μm SU-8 2015 negative epoxy photo-resist was deposited on the wafer via spin coating. A subsequent soft bake at 95 °C for 4 min flashed the solvent of the SU-8, resulting in a hardened and solidified state. The UV exposure of the channel geometry mask was carried out with the epoxy developing step before a post-exposure bake at 95 °C for 5 min to cross-link the desired geometrical structure.

The SU-8 structure fabricated on the silicon wafer served as a casting master. To create hydrophilic surfaces with a contact angle of less than 2 degrees, 1% by volume of polyethylene oxide (PEO) was mixed with the PDMS. After degassing the mixture in a vacuum chamber, the PDMS–PEO mixture was poured into the casting tray containing the silicon wafer master, which was cured in an oven at 80 °C for 2 h.

The tops of the individual PDMS chips were covered with glass microscope coverslips. This enclosed the microchannel, leaving only the inlet and outlet open to air, and also resulted in a hydrophilic top channel surface.

### 2.4. Experimental Analysis of Hematological Diagnostics Using Microfluidic Chips

A drop of whole blood was mixed in a 1:1 ratio with 40 μg/mL Acridine Orange in PBS and placed on the chip inlet. The mixture was pulled by capillary forces to the field of view, where it was illuminated and a fluorescent image was captured, as depicted in Figure 1. This shows the potential applications of whole blood processing, particularly of investigating blood cells optically in rectangular channels, while having filtering-free and efficient capillary pumping systems.

## 3. Results and Discussion

To reduce the analysis time of a 2 μL sample volume, the Ansys simulations were conducted using various chip pillar shapes to identify the designs with the fastest filling times.

### 3.1. Water Simulation

**Figure 4:**
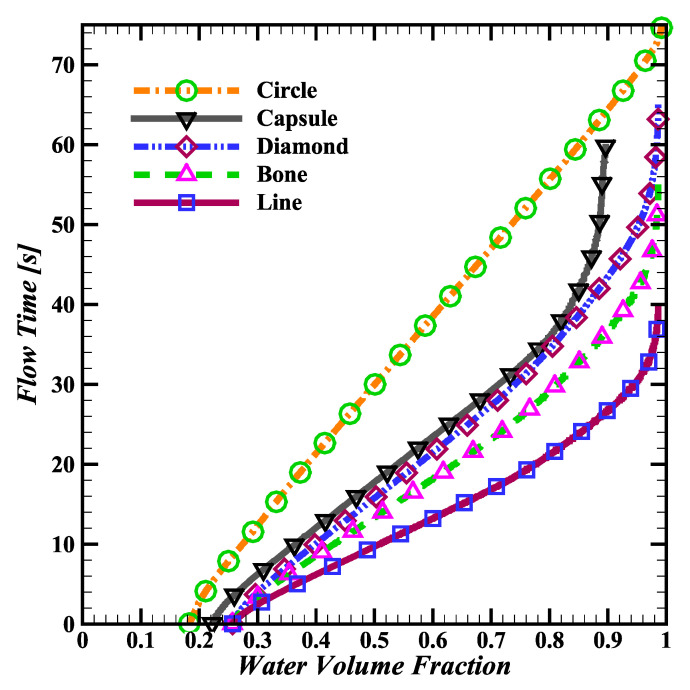
Simulated water filling time for chip pumps with either circle, diamond, capsule, bone, or line pillar shapes from Figure 3. Non-circular pillar shapes exhibit a non-linear flow rate, slowing as the water volume fraction increases.

Figure 4 compares the simulated time for water to fill chips created with each of the five different pump pillar shapes illustrated in Figure 3. The slight variation in the plotted initial water volume fraction stems from the cumulative area difference for the different pillar shapes within the capillary pumps.

Based on the simulations, line-shaped pillars have the fastest filling time of just 40 s, followed by bone-shaped pillars at 55 s, diamond-shaped at 65 s, and circular-shaped pillars at 75 s. After about 60 s, the capsule-shaped pillars saturate at a 90% water volume fraction and the chip does not fill completely. All non-circular pillar shape simulations exhibit slowing flow rates as the chip fills up.

**Figure 5:**
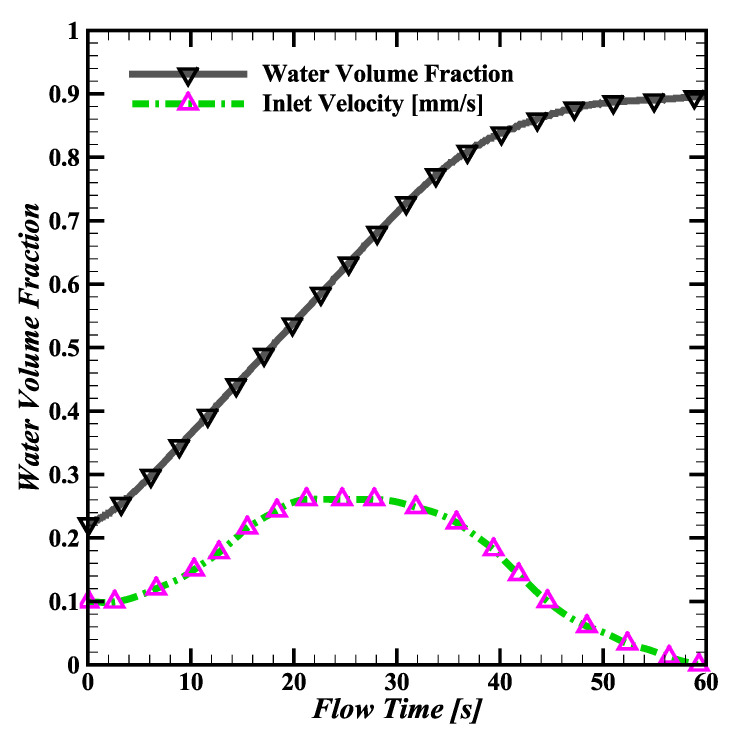
Simulated inlet velocity for capsule-shaped pillars. For the first 20 s, the water velocity steadily increases from the 0.1 mm/s initial condition. Once the chip reaches 60% capacity, the velocity decreases until the flow stops at 90% capacity, never filling to 100%.

Figure 5 shows the chip inlet velocity as a function of time for the capsule-shaped pillars. From the initial condition of 0.1 mm/s, the inlet velocity steadily rises for the first 20 s, until the chip is 60% filled. The velocity begins to steadily decrease until a 90% water volume fraction is reached, and the flow stops. With this pillar shape, the viscous forces in the pump overpower the capillary forces before the chip is completely filled.

### 3.2. Comparison of Water and Blood Plasma Simulations

**Figure 6:**
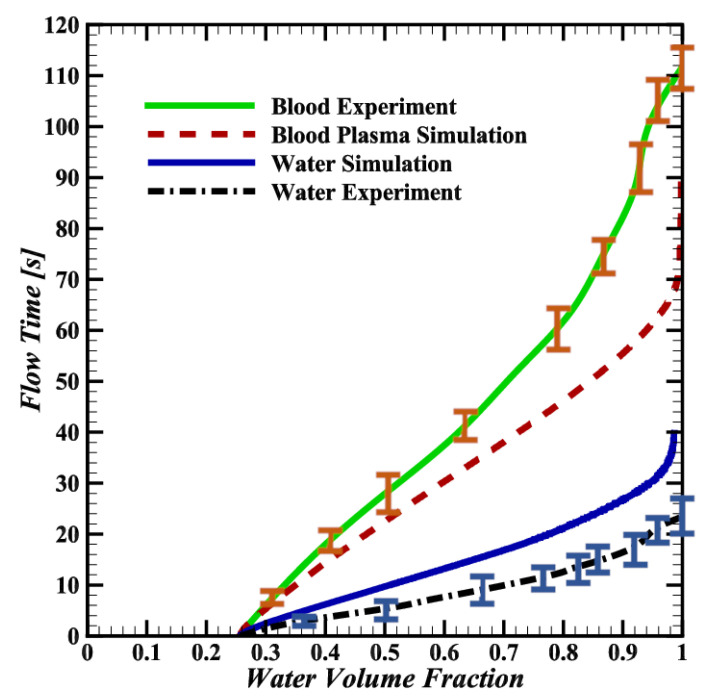
Simulated water and blood plasma as well as experimentally measured water and whole blood filling time in line-shaped pillar structures. Error bars indicate the standard deviation of the 10 repetitions performed for each fluid. For both the simulation and experiment plots, at (t=0 s), 25% of the chip’s volume is filled with fluid, coinciding with Figure 2.

The simulations of the line-shaped pillars showed the greatest potential to speed up chip filling. The simulation results were benchmarked against 10 repeated experiments using water and 10 experiments using blood, where experimental volume fractions were obtained by tracing the fluid coverage from images captured during filling. Comparison plots are shown in Figure 6.

The line-shaped pillar simulations result in filling times of 35 s for water and 70 s for blood plasma, whereas the experiments result in average filling times of 24 s for water and 111 s for blood. For both the simulations and experiments, chip filling occurs more slowly with blood plasma, as would be expected due to it being over three times more viscous than water. Interestingly, while the experimental filling rate appears to slow with higher volume fractions, in line with the simulations, the strongly asymptotic slowing seen in the simulations is not observed.

### 3.3. Discrepancies between Simulations and Experiments

Despite the modeling simplifications used, the simulated water filling time is faster but closely tracks that in the experiments. As depicted in Figure 6, differences between the simulated and experimentally observed pump filling have been noted. This discrepancy arises from the assumption made in the simulation that the contact angle is identical for all surfaces. In reality, the channel has a glass top surface and PDMS walls, with correspondingly different surface tension. Additionally, variability in the surface roughness along the PDMS channels was observed, caused by the peeling of the PDMS from the silicon and epoxy master structure. The variable surface of a real PDMS chip is challenging to capture in a simulation, and the divergence between the simulation and experimental results with water can be attributed to the difference in surface tension between the simulated and actual materials.

**Figure 7:**
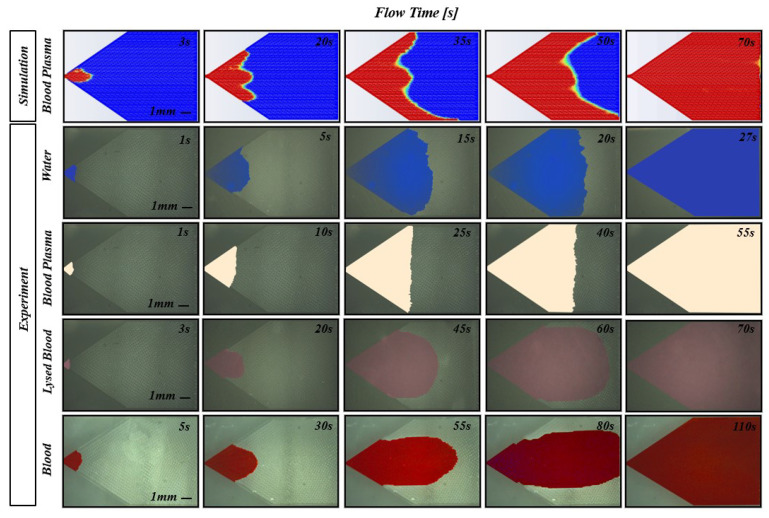
Comparison of capillary pump filling patterns over time. From top to bottom: the filling patterns for simulated blood plasma, followed by the experimental results for water, blood plasma, lysed blood, and whole blood. From left to right: the flow pattern’s evolution with time. Due to the different flow rates observed between cases, comparable volume fractions occur at different times.

In the experiments, whole blood shows a dominant axial diffusion component, as seen in the last row of Figure 7, compared to the more diffuse pattern of the blood plasma in the simulations. Moreover, the simulated filling time for blood plasma is about 40 s (35%) slower than the whole blood experimental results. The observed discrepancies in both the flow pattern and filling time in Figure 7 stem from the RBCs not being modeled in the blood flow simulations.

Figure 7 reveals a 15 s discrepancy between the simulated and experimental blood plasma behavior, due to the simulation’s constant surface tension assumption, which contrasts the variable surface tension exhibited by materials like PDMS and glass in contact with blood plasma.

When blood flows through narrow channels, erythrocytes tend to flow in the middle, leaving blood plasma near the walls and lowering the apparent viscosity of the flowing blood. This phenomena, the Fåhræus–Lindqvist effect [58], becomes more pronounced in narrower channels. In microfluidic structures separated by 50 μm, such as in our microfluidic pumps, the fluid properties can substantially deviate from the macroscopic values shown in Table 1. Furthermore, the inertia of the RBCs flowing through the capillary pump was presumed to be the primary driver of the axially diffusing flow of the whole blood experiment in Figure 7. To experimentally approximate homogeneous blood and minimize the influence of cell bodies on the flow, experiments were conducted with blood plasma and blood mixed with a lysis buffer to break down the RBC membrane.

Blood plasma is the primary constituent of whole blood and the main component interacting at the channel boundaries. By testing it in isolation, the viscous and surface tension effects experienced by whole blood without the influence of RBC inertia could be approximated. Compared to the whole blood experiment depicted in Figure 7, isolated blood plasma showed no dominant axial diffusion and had a fluid front similar to that of water. The chip filling time is slower than with water, owing to the higher viscosity of blood plasma. The marked difference in the flow pattern and filling time observed between the whole blood and blood plasma supports the premise that cells in the blood play a significant role in the filling pattern and chip filling time.

Finally, the whole blood was lysed with a 1X RBC lysis buffer at a 1:10 blood-to-buffer ratio, as per the manufacturer’s instructions. The fluid properties of the resulting mixture were not representative of whole blood due to the contents of the RBCs being released into the fluid upon rupturing and the dominant mixture component being the buffer solution and not blood. Interestingly, the filling pattern in Figure 7 appears to be a hybrid between those of blood plasma and whole blood. This can be attributed to the cell membranes of the lysed cells and possibly a small fraction of un-lysed cells affecting the flow in a similar but less pronounced way compared to whole blood.

## 4. Conclusions

This project aimed to optimize the whole blood flow within a microfluidic chip to achieve rapid filling times without the use of active pumps for PoC applications. Microfluidic capillary pillar structures were investigated that could draw 2 μL of whole blood through a 250 μm × 30 μm PDMS–PEO channel cross-section in under 2 min for the imaging and analysis of various cells.

Simulations with Ansys Fluent were used to identify the optimal capillary pump pillar shapes, which were verified with experiments. While the simulations agreed with the general trend of the experimental results, the pump filling time and flow pattern for the blood plasma flow were not fully consistent with the whole blood experimental results. To make the simulations computationally feasible, the blood plasma was assumed to be homogeneous and Newtonian, and the channel surfaces were assumed to have homogeneous wettability. These assumptions, particularly those neglecting the influence of the red blood cell momentum on the flow and the Fåhræus–Lindqvist effect, were likely the cause of the modeling discrepancies. Nonetheless, our results can be a useful aid in designing passive microfluidic pumps by quantifying the efficiency of different pump pillar shapes on the fluid flow.

## Figures and Tables

**Table 1:** Fluid properties of water, blood plasma, and air used for simulations.

Physical Property	Water (H_2_O)	Blood Plasma	Air
Density (kg/m^3^)	998.2	1080	1.1614
Dynamic Viscosity (mPa·s)	1.003	3.5	0.0185
Surface Tension (N/m)	0.0728	0.06	–

## Data Availability

Data is contained within the article.

## Abbreviations

The following abbreviations are used in this manuscript:

CFD	Computational Fluid Dynamics
AO	Acridine Orange
CSF	Continuum Surface Force
PDMS	Polydimethylsiloxane
PEO	Polyethylene Oxide
PoC	Point-of-Care
RBC	Red Blood Cell (Erythrocyte)
VOF	Volume of Fluid
WBC	White Blood Cell (Leukocyte)
WHO	World Health Organization
